# Effect of a short questionnaire (7VTE-Quest) on venous thromboembolism to guide education during consultation

**DOI:** 10.1097/MD.0000000000050491

**Published:** 2026-09-18

**Authors:** Thibaut Sarger, Helene Desmurs-Clavel, Iman Melin-Boucetta, Yesim Dargaud, Judith Catella

**Affiliations:** a Service de Médecine Interne, Hôpital Edouard Herriot, Hospices Civils de Lyon, Lyon, France; b GEMMAT, Groupe d’Etude Multidisciplinaire en Maladies Thrombotiques, Lyon, France; c Laboratoire Interuniversitaire de Biologie de la Motricité, Université Claude Bernard Lyon 1, Université de Lyon, Lyon, France; d Unité d’hémostase Clinique, Hôpital Cardiologique Louis Pradel, Hospices Civils de Lyon, Lyon, France.

**Keywords:** anticoagulant, patient education, patient knowledge, venous thromboembolism

## Abstract

Anticoagulation treatment of venous thromboembolism (VTE) carries potential risks, including major bleeding and recurrent VTE, often due to poor adherence, which is itself associated with insufficient patient knowledge. To investigate patient knowledge over time to evaluate the effectiveness of the strategy of patient education employed in our clinic. We created a short patient questionnaire (7VTE-Quest), completed in the waiting room and used to guide patient education during consultation. Patients requiring anticoagulant therapy ≥3 months and having completed the 7VTE-Quest at 2 different consultations between March 2022 and December 2024 were included. The primary endpoint was improved score (≥1 point) at second completion of the questionnaire. A total of 135 patients completed the questionnaire a second time a mean (±SD) 211 (±169) days after the initial completion. After excluding 4 patients who achieved a perfect score both times, the score improved for 56.5% (74/131) of patients. The change in total score ranged from −3 to +4. The greatest overall gain was for questions on management of forgotten medication dose (20.0%, 27/135) and interaction between anticoagulants and NSAIDs (11.1%, 15/135). A regression in the number of patients providing correct answers was observed for all questions between 1.6 and 40%. The greatest regression was observed for NSAID contraindication: 40.0% (14/35) of patients could no longer recall this contraindication, while 29.0% (29/100) of those who could not initially recall this contraindication did so at the second completion. The strategy we propose shows an improvement of patient knowledge on their VTE disease and treatment but failed to assess its impact on clinical outcomes.

**Key Points**

- Major bleeding and recurrent VTE under anticoagulant are associated with poor patient knowledge.
- Patients completed 7 questions in the waiting room to guide the education during consultation.
- At the second completion of the questionnaire, 57% of 131 patients increased their score.
- A regression of correct answer was observed for all questions.

## 1. Introduction

Venous thromboembolism (VTE), encompassing deep venous thrombosis (DVT) and pulmonary embolism (PE), is a widespread condition li**n**ked to substantial morbidity and mortality.^[1-3]^ Current European Society of Cardiology guidelines recommend anticoagulant treatment for ≥3 months, or indefinitely for unprovoked proximal DVT or PE.^[4]^ However, anticoagulation carries potential risks, including major bleeding^[5]^ and recurrent VTE, which are often due to poor adherence,^[6,7]^ which is itself associated with insufficient patient knowledge.^[8]^ It has been found that patient knowledge of VTE and anticoagulation is suboptimal.^[9]^ It has also been reported that >75% of VTE patients receive no information regarding the risks associated with anticoagulant**s**, long-term VTE complications, or recurrence prevention.^[10]^

To minimize complications and enhance anticoagulant therapy, some VTE centers provide structured educational programs for patients on anticoagulants.^[6,7,11]^ Establishing such programs is challenging due to caregiver time constraints, costs, and shorter hospital stays for VTE, as some patients are now managed as outpatients.^[12]^ There is no formal educational program after VTE in our hospital. Initial oral information given to patients can vary, especially as the initial management of VTE occurs at different sites. Consequently, we created a short patient questionnaire designed for practical use, as it includes only 7 questions (the 7VTE-Quest).^[13]^ The questionnaire is completed by patients before follow-up appointments in the waiting room; the answers guide the discussion with vascular physicians at our VTE clinic, allowing us to assess patient understanding of VTE and its treatment. This helps us target areas and patients needing further reinforcement. Other questionnaires have been developed and validated in VTE, but they have solely been used for a single evaluation of patient knowledge,^[9,14]^ as previously reported for the 7VTE-Quest.^[13]^ However, to evaluate the effectiveness of the strategy employed in our clinic on patient knowledge, we report herein the change in patient knowledge over time.

## 2. Materials and methods

The 7VTE-Quest questionnaire has been proposed since March 2022 to adult patients with a diagnosis of DVT or PE requiring anticoagulant therapy for ≥3 months, followed in our VTE clinic (part of the vascular medicine department of the Edouard Herriot university hospital, Lyon, France).

The diagnosis of PE is based on computed tomography scans. Lower limb DVT is diagnosed using ultrasonography and categorized as proximal or isolated distal DVT. The 7VTE-Quest questionnaire, previously described,^[13]^ is distributed at patient admission to the department before consultation; it is not distributed to patients with cognitive disorders or to those unable to complete it (e.g., paralysis or language barrier). The VTE knowledge assessment relied on the questionnaire and subsequent interviews in case of incorrect answer**s** to verify that the patient had understood the question.

For those who completed >2 questionnaires, only the first 2 were considered.

The primary endpoint was an improved score (≥1 point) at the second completion of the questionnaire. Secondary endpoints were to identify characteristics associated with score improvement, as well as characteristics associated with a poor VTE questionnaire score (<5, considering the median score in a previous study^[13]^).

Patient baseline characteristics (age, sex, tobacco use, active cancer, and other treatments; polymedication was defined as ≥3 other medications in addition to an anticoagulant) as well as data regarding VTE were collected from electronic medical files (Easily; Hospices Civils de Lyon, Lyon, France). The index VTE was the VTE event leading to the first consultation in our VTE clinic, even if the patient had a previous VTE event. Data regarding VTE history and index VTE were collected. Data collected regarding the index VTE included its association with active cancer or with a transient major risk factor (immobilization, surgery, or hormonal exposure; according to guideline definition^[4]^) as well as hospital admission. In addition, bleeding history since the initiation of anticoagulation therapy for index VTE was collected. Bleeding was classified as major or non-major according to the International Society on Thrombosis and Hemostasis definition^[15]^; major bleeding was defined as any fatal bleeding or bleeding that caused a decrease of hemoglobin level in blood of 2 g/dL or which required a transfusion of at least 2 units of packed blood cells, or any hemorrhage into any major anatomical sites whether intracranial, peritoneal, pericardial, etc. Those who did not satisfy the criteria for major bleeding were considered to have non-major bleeding.

The study was approved by the institutional review board of the Hospices Civils de Lyon (number 23_5081); patient consent was not mandatory due to the retrospective nature of the study.

Descriptive variables are reported as means ± standard deviation (SD). Differences in mean values between groups were assessed using the Student *t*-test analysis of variance. Categorical variables were compared using the chi-squared test. Univariate analyses were performed to identify factors associated with score improvement between Q1 and Q2, as well as with a poor VTE questionnaire score at Q1. The predefined potential confounding factors tested were: characteristics of patients (age ≥ 55 years, sex), polymedication, tobacco use, active cancer, type of index VTE (PE [irrespective of DVT status]), first episode of VTE, association with a major risk factor for VTE, outpatient care), and bleeding history since anticoagulant treatment. In multivariate analysis, variables were selected on the following basis: *P*-value < .15 in univariate analysis, prevalence > 3%, clinical relevance, and <2% of missing data. Factors with a *P*-value < .05 in the multivariate analyses were considered independent confounding factors. All reported *P*-values are 2-sided, and a *P*-value < .05 was considered to be significant. Statistical analyses were performed using IBM® SPSS® Statistics version 21 (SPSS Inc., Chicago).

## 3. Results

### 3.1. First response to the 7VTE-Quest questionnaire (Q1)

A total of 356 patients filled out the questionnaire and subsequently consulted a vascular physician for their VTE, with personalized education provided based on their answers. The majority were men (54.4%) and the mean (±SD) age was 56.8 (±18.3) years. More than a third of patients were polymedicated (38.8%), 14.9% were active smokers, and 12.1% had concomitant active cancer (Table 1).

**Table 1:** Patient characteristics at first questionnaire completion (Q1).

	Total	VTE questionnaire score		
	Population	Score < 5	Score 5–7	
	n = 356	n = 152	n = 204	
Mean age, yr (SD)	**56.8 ± 18.3**	**62.5 ± 18.5**	**52.6 ± 17.1**	***
Age ≥ 55 yr, n (%)	**201 (56.5**)	**104 (62.8**)	**97 (47.5**)	***
Sex, female, n (%)	162 (45.6)	68 (44.7)	94 (46.1)	
Polymedication (≥3), n (%)	**136 (38.8**)	**80 (52.6**)	**56 (27.5**)	***
Current smoker, n (%)	53 (14.9)	20 (13.2)	33 (16.2)	
Active cancer, n (%)	43 (12.1)	22 (14.4)	21 (10.3)	
Index VTE				
PE ± DVT, n (%)	239 (67.1)	111 (73.0)	128 (62.7)	
First episod, n (%)	224 (62.9)	104 (68.4)	120 (58.8)	
Associated with major risk factor, n (%)	161 (45.2)	73 (48.0)	88 (43.1)	
Outpatient care, n (%)	**135 (37.9**)	**41 (27.0**)	**94 (46.1**)	***
Bleeding history on anticoagulant				
Major bleeding, n (%)	9 (2.5)	5 (3.3)	4 (1.9)	
Non-major bleeding, n (%)	49 (13.8)	23 (15.1)	26 (12.7)	
Intervalle Q1-index VTE, d	301 ± 572	273 ± 552	322 ± 587	

Bold values indicate statistical significance.

DVT = deep venous thrombosis, PE = pulmonary embolism, Q1 = first questionnaire completion, SD = standard deviation, VTE = venous thromboembolism.

*** *P*-value < .001.

For more than two-thirds of patients, the index VTE was PE (irrespective of DVT status; 67.1%). More than one-third of patients had a first episode of VTE (37.4%), and for almost half of patients, the VTE was associated with a major risk factor (45.2%). More than a third of patients were outpatients (37.9%). Bleeding history since anticoagulation was reported in 16.0% of patients (2.5% major bleeding and 13.8% non-major bleeding; Table 1).

The questionnaire was filled out for the first time a mean (±SD) 301 (±572) days after index VTE. The total score was between 5 and 7 for 57.3% of patients, and <5 (poor score) for 42.7% (Table 1). In multivariate analysis, the factors significantly associated with a poor 7VTE-Quest score were age ≥ 55 years (OR 1.77, 95%CI 1.10–2.83) and polymedication ≥3 (OR 2.35, 95%CI 1.48–3.7; Table 2).

**Table 2:** Multivariable analysis of factors associated with poor VTE score (<5) at first questionnaire completion.

	OR (95% CI)	*P*-value
Age ≥ 55 yr	**1.77 (1.10–2.83**)	**.018**
Polymedication (≥3)	**2.35 (1.47–3.78**)	**<.001**
PE ± DVT	1.11 (0.66–1.88)	.657
Outpatient care	0.66 (0.39–1.10)	.108
First VTE episode	1.50 (0.94–2.41)	.091

References are age < 55 years, <3 medications, DVT only, inpatient care and recurrent VTE.

Bold values indicate statistical significance.

CI = confidence interval, DVT = deep venous thrombosis, OR = odds ratio, PE = pulmonary embolism, VTE = venous thromboembolism.

### 3.2. Second 7VTE-Quest questionnaire (Q2)

**Figure 1.**
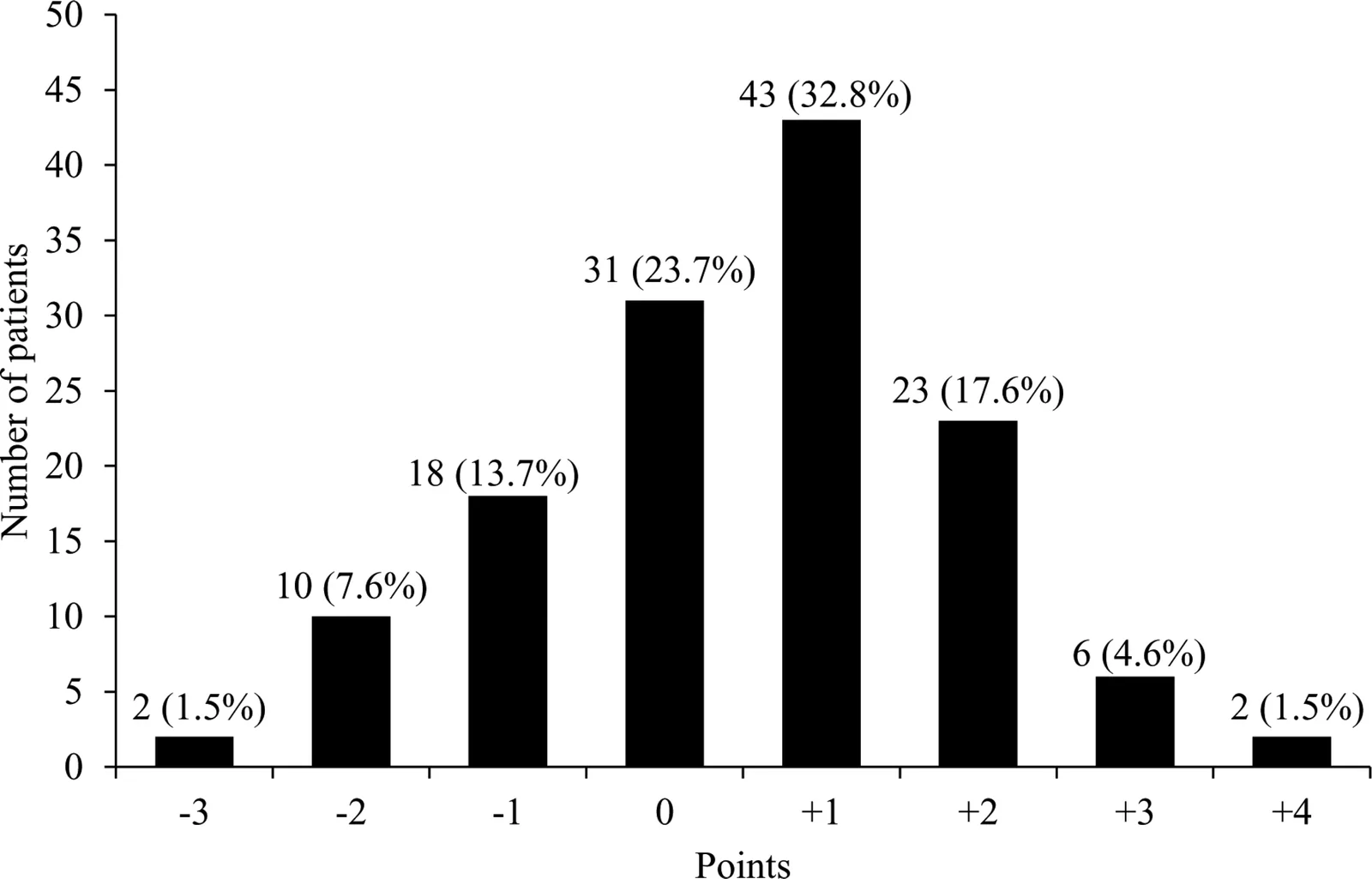
Change in score between first (Q1) and second questionnaire completion (Q2) (n = 131).

A total of 135 patients completed the questionnaire a second time a mean (±SD) 211 (± 169) days after the initial completion. During this interval, no patient experienced a VTE event; 20 (15.3%) experienced a non-major bleeding episode. The total score was between 5 and 7 for 72.6% (98/135) of patients, and <5 (poor score) for 27.4% (37/135). A total of 30/135 (22.2%) patients who initially had a poor score had a score between 5 and 7 at the second completion; the converse was found for 13/135 (9.6%) patients. The mean ± SD score significantly improved between the first (4.7 ± 1.4) and second (5.2 ± 1.4, *P* = .003) completion of the questionnaire. The change in score ranged from −3 to +4 (Fig. 1). Overall, and after excluding the 4 patients who achieved a perfect score both times, the score improved for 56.5% (74/131) of patients and did not improve (no change or lower) for 43.5% (57/131) of patients. The score worsened for 22.2% (30/135) of all patients included. There was no significant difference in patient characteristics according to whether or not the score improved (Table 3).

**Table 3:** Patient initial characteristics and outcomes between first (Q1) and second questionnaire completion (Q2).

	Total	Improvement	No improvement (no change or lower)	*P*-value
	n = 131	n = 74	n = 57	
Mean age, yr (SD)	57.4 ± 18.5	58.0 ± 19.2	58.4 ± 18.2	1
Age ≥ 55 yr, n (%)	79 (60.3)	45 (60.8)	34 (59.6)	1
Sex, female, n (%)	56 (42.7)	27 (36.5)	28 (49.1)	.16
Polymedication (≥3), n (%)	45 (34.4)	25 (33.8)	20 (35.1)	1
Current smoker, n (%)	24 (18.3)	16 (21.6)	8 (14.0)	.36
Active cancer, n (%)	22 (16.8)	11 (14.9)	11 (19.3)	.64
Index VTE				
PE ± DVT, n (%)	92 (70.2)	50 (67.6)	42 (73.7)	.56
First episode, n (%)	95 (72.5)	53 (71.6)	42 (73.7)	.85
Associated with major risk factor, n (%)	57 (43.5)	33 (44.6)	24 (42.1)	.86
Outpatient care, n (%)	49 (37.4)	26 (35.1)	23 (40.4)	.59
Outcomes (between Q1 and Q2)				
Major bleeding, n (%)	0	0	0	
Non-major bleeding, n (%)	20 (15.3)	11 (14.8)	9 (15.8)	1
Recurrent VTE, n (%)	0	0	0	
Mean interval Q1–Q2, days ± SD	211 ± 169	203 ± 165	216 ± 154	.63

The 4 patients with a perfect (7/7) score at Q1 and Q2 were excluded from this analysis.

DVT = deep venous thrombosis, PE = pulmonary embolism, Q1 = first questionnaire completion, Q2 = questionnaire second completion, SD = standard deviation, VTE = Venous thromboembolism.

At the second completion, the three most infrequently correctly answered questions concerned the risk of bleeding with anticoagulants (63.7%, 86/135), activities to avoid with anticoagulants (63.7%, 86/135), and knowing the interaction between anticoagulants and NSAIDs (37.0%, 50/135). The proportion of patients aware that they had VTE remained the same (92.6%); for all other questions, the frequency of correct response was higher when patients completed the questionnaire for the second time. The greatest overall gain was observed for the questions on management of forgotten medication dose (20.0%, 27/135) and the interaction between anticoagulants and NSAIDs (11.1%, 15/135). Among those who were initially unable to explain the disease, 69.7% (23/33) were aware of this at the second questionnaire completion; conversely, 12.7% (13/102) of those initially able to explain the disease were unable to do so at second completion. Among those who could not initially recall the contraindication for NSAIDs, 29.0% (29/100) could recall this, while 40.0% (14/35) could no longer recall this contraindication; similarly, 57.1% (40/70) of patients became aware of the management of a forgotten medication dose, while 21.5% (14/65) were no longer aware of this (Table 4).

**Table 4:** Change in answers between first (Q1) and second questionnaire completion (Q2).

	Q1		Q2			
N = 135	Correct answer	Incorrect answer	Correct answers	Incorrect answer	Relative improvement Q1–Q2	Relative regression Q1–Q2
Knows disease	125 (92.6)	10 (7.4)	125 (92.6)	10 (7.4)	4/10 (40)	4/125 (3.2)
Explain disease	102 (75.6)	33 (2.4)	112 (83.0)	23 (17.0)	23/33 (69.7)	13/102 (12.7)
Knows treatment	126 (93.3)	9 (6.7)	128 (94.8)	7 (5.2)	4/9 (44.4)	2/126 (1.6)
Knows bleeding risk	76 (56.3)	59 (43.7)	86 (63.7)	49 (36.2)	25/59 (42.4)	15/76 (19.7)
NSAIDs interaction	35 (25.9)	100 (74.1)	50 (37.0)	85 (70.0)	29/100 (29.0)	14/35 (40.0)
Forgotten medication	65 (48.1)	70 (51.9)	91 (67.4)	44 (32.6)	40/70 (57.1)	14/65 (21.5)
Activities to avoid	78 (57.8)	57 (42.2)	86 (63.7)	49 (36.3)	21/57 (36.8)	13/78 (16.7)

Relative improvement to incorrect answers at Q1; relative regression to correct answers at Q1.

NSAIDs = non-steroidal anti-inflammatory drugs, Q1 = first questionnaire completion, Q2 = second questionnaire completion.

## 4. Discussion

Herein, just over half of patients who filled out the 7VTE-Quest questionnaire, which was used to guide consultations with a vascular physician, had significantly improved knowledge of VTE disease and treatment. Increasing the precision with a larger cohort compared to our previous study,^[13]^ we confirm that initial poor knowledge was associated with age ≥55 years and also found an association with polymedication (≥3 drugs). However, the strategy we proposed, with a questionnaire and personalized education during subsequent consultation, showed improved patient knowledge to the same extent irrespective of the patient’s initial characteristics. Furthermore, although it is of note that the greatest knowledge gains were observed for the ability to explain the disease and the management of a forgotten medication dose, the results concerning regression in patient knowledge over time are of equal importance. The latter underlines the need for repeated evaluation to identify the current knowledge of patients and provide tailored education.

The most important regression was observed for the question concerning interaction between anticoagulants and NSAIDs, with almost half of patients who were initially aware of this losing this knowledge at follow-up, and the lowest relative improvement was found for this question. This means patients who have this knowledge are likely to lose it over time, and it is difficult for them to acquire it. More generally, this question was answered correctly least frequently at the second questionnaire completion (just over a third of patients). This is a critical area to address because the combination of NSAIDs and anticoagulation significantly increases the risk of major bleeding (hazard ratio [HR] 2.77, 95% CI: 1.84–4.19) and also the risk of stroke (HR 3.01, 95% CI: 1.63–5.55).^[16]^ Studies have reported that nearly a quarter of patients on vitamin K antagonists^[17]^ and 9% of those receiving apixaban^[18]^ are exposed to NSAIDs. A concerning finding is that less knowledge about over-the-counter products with potentially serious interactions was associated with greater over-the-counter product use (OR 0.54, 95% CI: 0.35–0.85),^[19]^ highlighting the importance of patient education on this subject. To address these vulnerabilities, targeted educational reinforcement may be required, including the systematic delivery of focused messages on high-risk drug interactions, the use of simplified visual aids, and the integration of standardized reminder materials during follow-up visits. Incorporating brief reinforcement at each consultation, combined with written or digital take-home supports, may help improve retention of safety-critical information. However, the implementation of such structured and repeated educational strategies inevitably requires dedicated time and trained personnel, resources that are often limited or unavailable in routine clinical practice. Other pedagogical methods could be employed. For example, a model featuring animation of the kidney and its function demonstrated its effectiveness in improving patient knowledge on the risk of acute kidney injury from NSAIDs.^[20]^ Furthermore, countries such as the United Kingdom and Switzerland provide patients on anticoagulants with an anticoagulant card. This card alerts patients to the necessity of consulting healthcare professionals before taking over-the-counter medications. In France, while patients find a patient card in the medication box, this card does not specifically warn about interaction risks. Therefore, adding this warning in France and other countries where it is not currently indicated on the patient card could be highly beneficial.

At the second completion of the questionnaire, more than a third of patients included in the present study were unaware of the risk of bleeding and, consequently, which activities at risk of bleeding to avoid. Interestingly, we found no association between bleeding experience and patient knowledge. This aligns with that reported by Casais et al, who observed that hemorrhage experience did not modify patient’s perception of bleeding risk.^[21]^ Moreover, Golab et al reported that a higher risk of bleeding, as determined by the VTE-BLEED score, was related to a lower overall baseline score on the Jessa Atrial Fibrillation Knowledge Questionnaire adapted for VTE.^[22]^ The authors also found that poor knowledge was associated with a higher risk of major bleeding during follow-up; however, this could potentially be a consequence of an initial higher bleeding risk. Although there remains room for improvement, the strategy seems to have a positive effect, and we hope that over the longer term the proportion of patients aware of these risks will increase.

This study, due to its retrospective design, presents major limitations, primarily the inability to control for several important confounding factors that may have significantly influenced the results.^[23]^ In particular, patients’ level of education, health literacy, and other sources of knowledge acquisition (such as prior medical counseling, media exposure, or self-directed learning) could not be systematically assessed or adjusted for. These unmeasured variables are known to have a substantial impact on medical knowledge and may have strongly affected baseline VTE knowledge as well as its evolution over time. As a result, the observed changes cannot be attributed with certainty to the intervention alone, thereby limiting the internal validity and interpretability of our findings. This methodological constraint inherent to the retrospective design should be considered a central limitation of the present study.

Furthermore, the VTE knowledge assessment relied on a questionnaire and subsequent interviews. The vascular physician’s discretion in conducting the interview might have introduced variability and subjectivity to scoring, and thus influence the final results. Furthermore, the final results reflect education during the last consultation but also information gathered by the patient from other sources. This is, however, not a concern for the present study as our objective is the improvement of patient knowledge, irrespective of the source of education. The mean duration between the first and second questionnaire completions was approximately 7 months. This may be considered long and could have participated in limiting improvement of knowledge and increasing regression, but it does reflect real-life practice. Finally, the limited sample size completing the questionnaire for the second time is a notable constraint. Furthermore, the VTE questionnaire was not distributed to all patients from our clinic, particularly those with cognitive impairments who likely have poor VTE knowledge. This could lead to an underestimation of the true prevalence of poor VTE knowledge and associated factors.

## 5. Conclusion

The strategy we propose shows an improvement in patient knowledge of their VTE disease and treatment. Due to an insufficient number of outcome events, no definitive conclusions could be drawn regarding the clinical impact of improved patient knowledge. Further research is needed to investigate the relationship between the improvement in patient knowledge achieved by this strategy and the rates of VTE recurrence and bleeding.

## Author contributions

**Conceptualization:** Yesim Dargaud, Judith Catella.

**Data curation:** Thibaut Sarger, Helene Desmurs-Clavel, Iman Melin-Boucetta, Judith Catella.

**Formal analysis:** Thibaut Sarger, Judith Catella.

**Investigation:** Thibaut Sarger, Judith Catella.

**Methodology:** Judith Catella.

**Validation:** Judith Catella.

**Writing – original draft:** Thibaut Sarger, Yesim Dargaud, Judith Catella.

**Writing – review & editing:** Judith Catella.
